# Biomolecule from *Trigonella stellata* from Saudi Flora to Suppress Osteoporosis via Osteostromal Regulations

**DOI:** 10.3390/plants9111610

**Published:** 2020-11-20

**Authors:** Hairul-Islam Mohamed Ibrahim, Hossam M. Darrag, Mohammed Refdan Alhajhoj, Hany Ezzat Khalil

**Affiliations:** 1 Biological Sciences Department, College of Science, King Faisal University, Al-Ahsa 31982, Saudi Arabia; 2 Pondicherry Centre for Biological Sciences and Educational Trust, Kottakuppam 605104, India; 3 Research and Training Station, King Faisal University, Al-Ahsa 31982, Saudi Arabia; hdarag@kfu.edu.sa; 4 Pesticide Chemistry and Technology Department, Faculty of Agriculture, Alexandria University, Alexandria 21545, Egypt; 5 Arid Land Agriculture Department, College of agricultural and Food Sciences, King Faisal University, Al-Ahsa 31982, Saudi Arabia; malhajhoj@kfu.edu.sa; 6 Department of Pharmaceutical Sciences, College of Clinical Pharmacy, King Faisal University, Al-Ahsa 31982, Saudi Arabia; heahmed@kfu.edu.sa; 7 Department of Pharmacognosy, Faculty of Pharmacy, Minia University, Minia 61519, Egypt

**Keywords:** *Trigonella stellata*, caffeic acid, osteoporosis, osteoblast, osteoclast, BMP

## Abstract

*Trigonella stellata* has used in folk medicine as palatable and nutraceutical herb. It also regulates hypocholesterolemia, hypoglycemia, and has showed anti-inflammatory activities as well as antioxidants efficacy. Osteoporosis is a one of bone metabolic disorders and is continuously increasing worldwide. In the present study, caffeic acid was isolated from *Trigonella stellata* and identified using 1 D- and 2 D-NMR spectroscopic data. Caffeic acid was investigated on osteoblast and osteoclast in vitro using mice bone marrow-derived mesenchymal cells. Caffeic acid played reciprocal proliferation between osteoblast and osteoclast cells and accelerated the bone mineralization. It was confirmed by cytotoxicity, alkaline phosphatase (ALP), alizarin red S (ARS), and Tartrate resistant acid phosphatase (TRAP) assay. Caffeic acid regulated the osteogenic marker and upregulated the osteopontin, osteocalcin, and bone morphogenic proteins (BMP). Quantitative real time PCR and Western blot were used to quantify the mRNA and protein markers. It also regulated the matrix metalloprotease-2 (MMP-2) and cathepsin-K proteolytic markers in osteoclast cells. In addition, caffeic acid inhibited bone resorption in osteoclast cells. On the other hand, it upregulate osteoblast differentiation through stimulation of extracellular calcium concentrations osteoblast differentiation, respectively. The results also were confirmed through in silico docking of caffeic acid against cathepsin-B and cathepsin-K markers. These findings revealed that caffeic acid has a potential role in bone-metabolic disorder through its multifaceted effects on osteoblast and osteoclast regulations and controls osteoporosis.

## 1. Introduction

Osteoporosis is a metabolic bone disease characterized by low bone mass, imbalanced bone cell types that leads to osteoarthritis (OA). Bone is a metabolically active connective tissue, ability to regenerate from the incidence and accident of fractures [1]. Bone reabsorption and osteogenic formation tends the remodeling of bone loss and balancing the bone stereotypes. Osteoclast types of cells efficiently absorb the damaged bone cells and promote osteoconductivity, as in-growth around the bone; induction of osteogenic response promote progenitor cell differentiation and mineral storage in osteoblastic lineages. Balances between osteoblast and osteoclast would be happened by bone marrow derived cells. Bone-marrow derived mesenchymal stem cells (BM-MSCs) are multipotent, differentiated into connective tissue, and develop into mature osteoblasts [2]. Mesenchymal cells directly involved in extracellular matrix composition, mineralization, and coordinate the differentiation of osteostromal cells [3]. Osteoclasts are generated from precursor cells in presence of receptor activator of nuclear factor kappa-B ligand (RANKL) [4]. These inductions activate the inflammatory mediators which given back bone loss. Bone morphogenetic protein (BMP) and IL-10 are examples of osteogenic markers widely used in differentiation of mesenchymal stem cells into osteoblasts. Mitogen activated protein kinase (MAPK) regulates the osteoblast-specific transcription factors for differentiation process [5].

Simple analgesics are now recognized as one of the first line pharmacological treatment of uncomplicated OA. Whereas some non-steroidal anti-inflammatory drugs (NSAIDS) may show some fatal adverse effects particularly if used in long term treatment plans.

Natural products have been recently considered as a source of important therapeutic candidates, that could treat various diseases and considered effective for maintaining good health [6]. The interest in drugs derived from plants is predominantly attributed to the trust that green medicine is safe and dependable in comparison to the synthetic one. Wide ranges of natural candidates have implemented in treatment of chronic and infectious diseases [7].

Currently, a plethora of agents are available for the treatment of inflammatory disorders including OA, but some of the drugs are associated with risk of life-threatening adverse effects leading to its withdrawal from market [8]. Hence, the management of inflammatory disorders using medicines without side effects is still a challenge. In the last decades, hundreds of reports were published regarding the anti-inflammatory activities of plants that were available for alternative therapy [9]. Particularly, phenolic bioactive constituents showed significant attention due to their modulatory activities on inflammasomes [10]. Therefore, it is highly required to find out herbal products or nutraceuticals, which can be used as add-on therapy for long-term management of inflammatory disorders.

Studies have proven that leguminous plants may act as reservoirs of potential secondary metabolites of diverse therapeutic utilities and can produce an anti-inflammatory effect as well as they have significant nutritional value [11,12]. In this aspect, there is a consumption of *Trigonella* species (member leguminous herbs) because of their nutritional value. Particularly, *Trigonella foenum-graecum*, which is a small plant with several benefits, attributed to the diverse array of phytoconstituents such as phenolics and flavonoids [13]. Traditionally *Trigonella foenum-graecum* is reputed to exert anticancer, antidiabetic, antioxidant, antihyperlipidemic, and other various pharmacological effects [14].

*Trigonella stellata* (*T. stellate*) (Leguminosae) is a member of the genus *Trigonella* that is native and very common to grow wildly in Arabian Peninsula including Saudi Arabia [15]. Traditionally, *T. stellata* is a palatable herb [16] and used to treat abdominal pain, diarrhea, dysentery [17], and as nutraceutical herb [18]. Recent studies reported that *T. stellata* contains isoflavans and saponins and showed antidiabetic and anti-hyperlipidemic activities [19,20].

No previous reports have been performed to illustrate the efficacy of *T. stellata* as anti-erosion agent in vitro and in vivo platforms. Therefore, this study was aimed to investigate the *T. stellata* for the first time for such activities.

## 2. Results

### 2.1. Isolation and Identification of Major Compound

**Figure 1:**
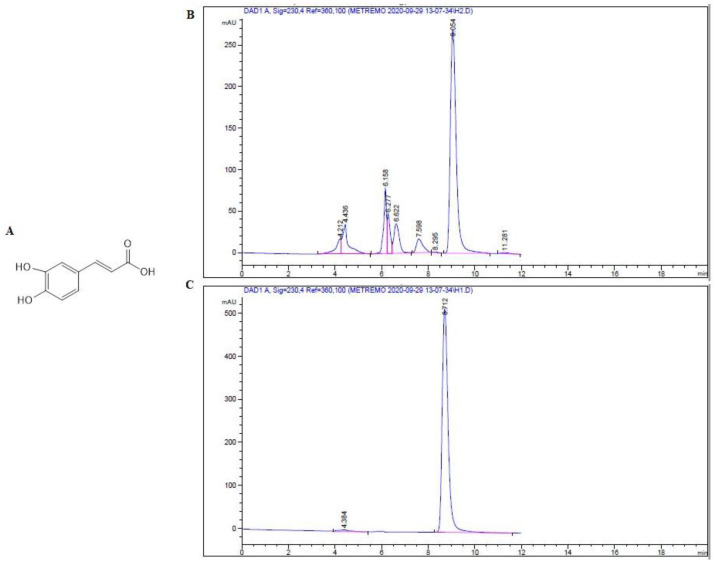
Structure of caffeic acid (CAF) isolated from *T. stellata* (**A**). HPLC chromatogram of SubFr. 2-4-4-1 (**B**). HPLC chromatogram of collected pure CAF (**C**).

The methanol extract of shade dried aerial parts (25.0 g) was subjected to several and repeated chromatographic techniques to give the pure phenolic compound; Caffeic acid (CAF) (23.6 mg) [21] (Figure 1). The structure was elucidated by inspection of 1 D- and 2 D-NMR spectroscopic data (Table 1 and Supplementary Material) and compared with literature values [21]. This study represents the first report on the isolation of CAF from *T. stellata*.

### 2.2. Effect of *T. Stellata* Extract Against Mesenchymal Cells

**Figure 2:**
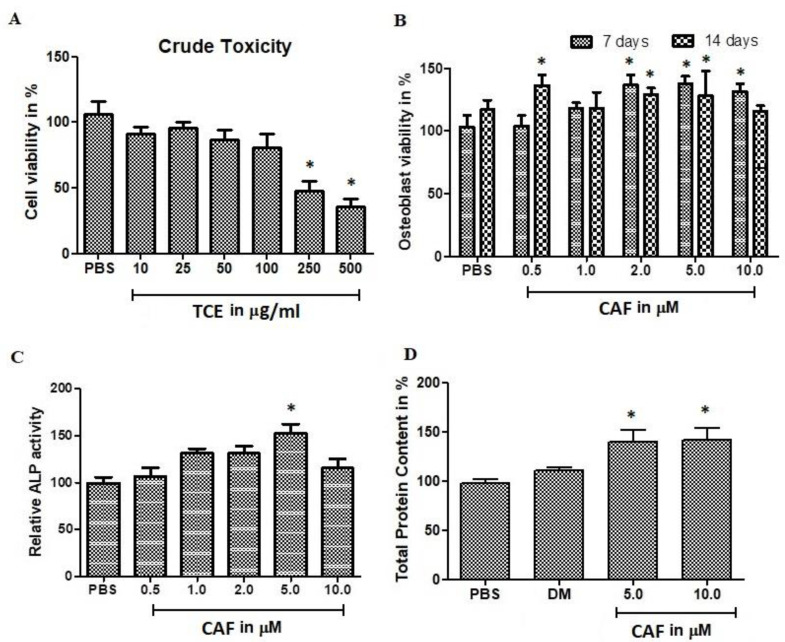
In vitro cytotoxic evaluation of *T. stellata* crude extract (TCE) and its metabolites. (**A**) The osteostromal cells were isolated from murine bone marrow and osteogenic characters were induced. The osteogenic cells were further analyzed for cytotoxic effect against *T. stellata* crude extract. The concentration tested from 10 μM to 500 μM. The crude extract was treated with osteogenic cells for 72 h and evaluate the cell viability using MTT reagent. (**B**) The major metabolites caffeic acid (CAF) was treated with osteogenic cells in concentration of 1.0 μM to 50 μM for 7 days and 14 days cultured cells. (**C**) Alkaline phosphatase-specific activity. (**D**) Total protein content. Bars represent the mean ± SD (*n* = 4). Statistical results are shown as * *p* < 0.05, Values compared between the PBS group with CAF at different concentrations.

The biocompatibility of *T. stellata* crude extract (TCE) against murine bone marrow derived mesenchymal cells. Initially, the TCE was evaluated using BM-MSCs viability assay at concentrations (10, 20, 50, 100, 250, and 500 μg/mL) by 3-(4,5-dimethylthiazol-2-yl)-2,5-diphenyltetrazolium bromide (MTT) method (Figure 2A) and neutral red (NR) assay (Figure 2B). This examination revealed that the TCE metabolites play a major role in cell biocompatible. The major moiety isolated from TCE was CAF. The selection of biocompatible concentrations of CAF was carried out using MTT assay. Cell viability was not inhibited up to 50 μg/mL of TCE concentration. The CAF at concentration >10 μM decreases the cell viability with an IC50 with >10 μM concentration. The neutral red assay estimates the uptake of colour from viable cells. NR measurements of the total viable cells was correlated with MTT assay. The NR results revealed that, insignificant cell death was observed at all the tested concentrations. Even at 10 μM CAF treatment showed <25% cell death.

Concerning, the effect of bone cell differentiation or proliferation, CAF significantly stimulated alkaline phosphatase (ALP) activity and bone cell differentiation in BM-MSCs cells. Thus, the effect of CAF on osteoblast culture from primary precursors cells was investigated. ALP was estimated at 14 days incubation (Figure 2C). The contents of ALP was increased 50% at 5-μM CAF treatments. ALP results were co-related with total protein content (Figure 2D). Total protein was raised up to 50% in CAF treated osteoblastic cells.

### 2.3. CAF Regulated In Vitro Mineralization of Osteoblastic Cells

**Figure 3:**
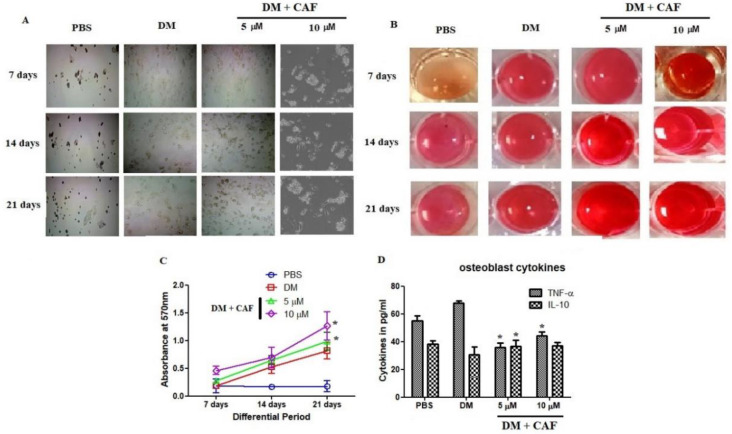
The differentiation and in vitro mineralization of osteoblast was evaluated at CAF treated conditions. (**A**) Representative macroscopic (**B**) Microscopic observation of alizarin red S (ARS) (**C**) ARS quantification for osteoblastic Differentiation at CAF treated conditions. (**D**) Cytokines estimation at 7th day of CAF treated osteoblastic cells. TNF-α and IL-10 were estimation using ELISA kits. Bars represent the mean ± SD (*n* = 4). Statistical results are shown as * *p* < 0.05. Values compared between the PBS group with CAF at different concentrations.

CAF regulated the bone cell differentiation and influenced the loading of calcium on osteoblastic cells. CAF stimulated the proliferation in osteogenic cells. Thus, the effect of CAF was investigated using in vitro osteoblastic cells primary stained with alizarin red S (ARS). As shown in Figure 3A,B, differentiated osteoblasts were stained with ARS for a period of 7, 14, and 21 days, respectively. The contents of ARS staining increased 100–200% at CAF (5 and 10 μM) Figure 3C. Whereas 10 μM showed highly significant amplification of loading mineralization as well as accelerates the ARS staining up to 2-fold compared to PBS group. The inflammatory cytokines from osteoblast differentiation were quantified and found CAF insignificantly regulated the IL-10 and it was not overexpressed by the differentiation cellular modifications Figure 3D. Whereas, TNF-α was reduced significantly at 5 μM of CAF treatment in osteoblast stereotypes.

### 2.4. Regulation of CAF on Osteoblastic Markers

**Figure 4:**
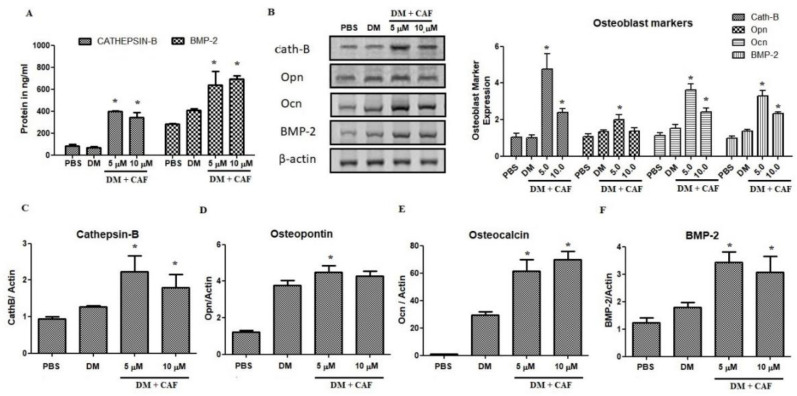
Effect of CAF on bone markers in osteoblast cell organization. (**A**) Cytokines estimation at 7th day of CAF treated osteoblastic cells. TNF-α and IL-10 were estimation using ELISA kits. (**B**) Effect of CAF on the protein expression of bone markers such as cathepsin-B (Cath-B), osteopontin (Opn), steocalcin (Ocn) and Bone morphogenic protein-2 (BMP-2). Protein expression were measured by Western blot analysis. (**C**–**F**) mRNA expression of bone markers on CAF treated osteoblastic cells and it was quantified by quantitative real time PCR was normalized to β-actin. (**C**) mRNA expression of cathepsin B, (**D**) mRNA expression of osteopontin, (**E**) mRNA expression of osteocalcin and (**F**) mRNA expression of BMP-2 marker, respectively. Bars represent the mean ± SD (*n* = 4). Statistical results are shown as * *p* < 0.05.

CAF assessed on mechanistic influences related to the effect on mineralization and differentiation. It was decided to examine the quantification of mRNA and protein markers related to osteoblastic regulations (Figure 4). The protein estimation of cathepsin-B and BMP-2 in CAF treated osteoblastic cells was shown in Figure 4A. The estimation showed that, cathepsin-B protein was significantly increased at 5 μM CAF treated osteoblastic cells. Moreover, BMP-2 was also upregulated parallel to Cath-B protein (Figure 4A). Differential media increased the BMP-2 level 60% compared to control and CAF treatment increased the level up to 300% compared to control groups. These results claim to investigate more markers related to osteoblastic cells. Immunoblot based estimation of cathepsin-B, osteopontin, osteocalcin, and BMP-2 were estimated in CAF treated cells. The protein of Cath-B, osteocalcin, and BMP-2 were increased significantly in CAF treated cells (Figure 4B). On the other hand, osteopontin was not upregulated by CAF treatments. Moreover, 10 μM of CAF treatment upregulate the transcript marker of osteoblast-related morphogenic proteins, osteocalcin (250%), as well as the osteo-inductive protein BMP-2 (230%) (Figure 4C–F). The significance observation was also noted in mRNA estimation of osteoblastic markers. Osteopontin was not significantly increased against differential medium (DM) group, but it was significant against control group.

### 2.5. Effect of CAF on Osteoclastogenic Regulations

**Figure 5:**
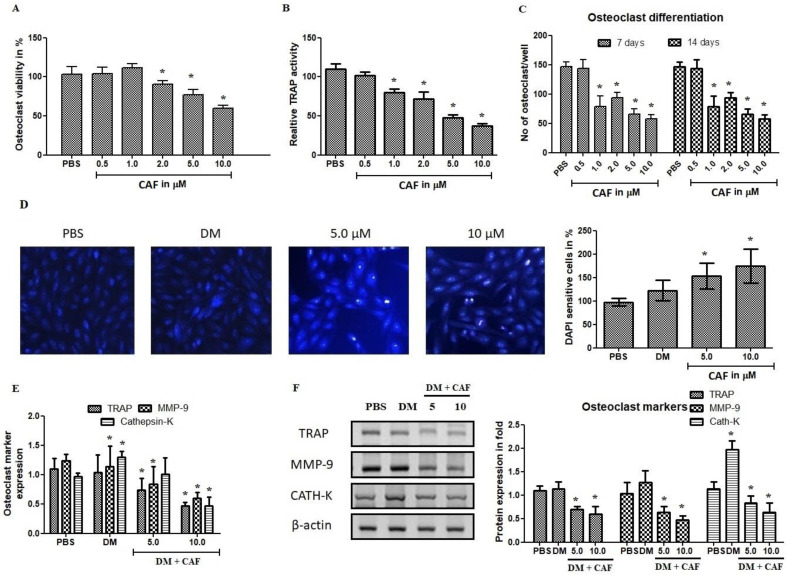
Effects of CAF on receptor activator of nuclear factor kappa-B ligand (RANKL)-induced osteoclast differentiation in mesenchymal cells. Bone-marrow derived mesenchymal stem cells (BM-MSCs) were cultured with vehicle or CAF (5 and 10 µM) in the presence RANKL (100 ng/mL) for 7 days. (**A**) Cultured cells tested against CAF treatment and analysed the cell viability. Cell viability of BM-MSCs was determined using the XTT assay (**B**) The estimation of Tartrate resistant acid phosphatase (TRAP) activity at 450 nm. (**C**) Cell differentiation effect was quantified on two intervals from 7 days to 10 days of incubation with CAF treatment. (**D**) RANKL induced osteoclast cells were treated with CAF and estimate the DAPI positive cells to confirm apoptotic induction as well as nuclear organization by fluorescence. Nuclei were stained by DAPI (blues signal). The area of DNA damage and nuclear organization was measured using ImageJ software. (**E**) Quantification of mRNA expression osteoclastic markers in CAF treated osteoclast cells using quantitative RT-PCR. The markers are TRAP, matrixmettalloprotease-9 (MMP-9), cathepsin-K (Cath-K). (**F**) Quantification of protein expression in osteoclastic cells treated with CAF using Immunoblot methods. The markers are TRAP, matrixmettalloprotease-9 (MMP-9), cathepsin-K (Cath-K). Bars represent the mean ± SD (*n* = 4). Statistical results are shown as * *p* < 0.05.

Osteoclasts are major cell type in bone mass and play a critical role in bone rejuvenation and resorption. The negative regulation of osteoclastogenesis has recognized to be a positive treatment for bone loss and bone degenerative diseases. Therefore, it was examined for the anti-osteoclastogenic effect using CAF in RANKL-induced osteoclast cells. As shown in Figure 5A, results showed that CAF treatment concentrations tested from 0.5 to 10 μM regulate the osteoclast cell viability. At 5 and 10 μM concentration plays a 30% and 45% cell death, respectively. CAF treatment strongly inhibited the tartrate resistant acid phosphatase (TRAP) positive osteoclast cells by a dose-dependent manner. The inhibition of osteoclast differentiation was confirmed by TRAP activity (Figure 5B). It was further evaluated using a differentiation platform with RANKL (Figure 5C). As shown in Figure 5D, CAF exhibited significant DNA damage to osteoclast cells. The inhibition was profound nuclear membrane organization and DNA linearization. These results claimed that CAF at 10 μM concentration inhibits the osteoclastogenesis and accelerates RANKL induced DNA damage. Apparently, it was further examined to confirm the osteoclastogenesis mRNA and protein markers.

Osteoclast marker investigation was done using mRNA and Western blot analysis. Western blot based quantification of TRAP, MMP-9, and cathepsin-K were estimated in CAF treated osteoclast cells. The significance observation was noted in mRNA estimation of osteoclast markers. TRAP expression was not appreciated in differential RANKL medium compared to control group, it was reduced at CAF 5 μM and 10 μM treated groups. The protein of TRAP, Cath-K, and MMP-9 were significantly decreased in CAF treated cells (Figure 5E,F). On the other hand, cathepsin-K was elevated at DM group compared to other tested groups. In addition, 10 μM of CAF treatment decreased the protein expression of MMP-9, these results revealed that CAF inhibit the migration and infiltration of bone cells. These results examined, showed that osteoclast markers were negative regulated by CAF treatment. It was significantly regulated by CAF compared to DM group. The inhibition of osteoclast genesis gives a remarkable output of *Trigonella* molecules reciprocally regulated the osteoblast and osteoclast cell types and might control the bone erosion and increases the reabsorption of senescent bone cells.

### 2.6. In Silico Docking of CAF against Cathepsin-B and Cathepsin-K Markers

**Figure 6:**
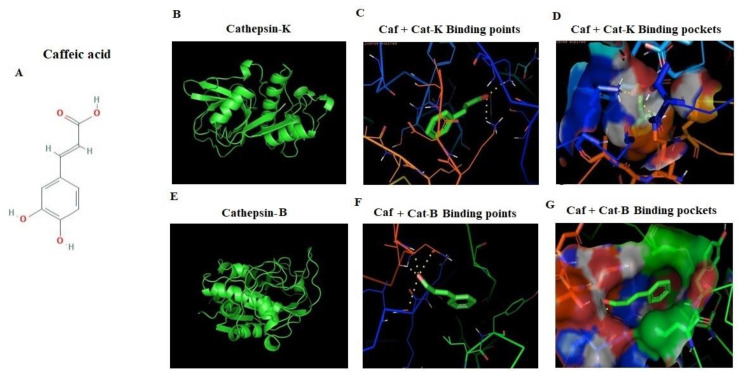
In silico interaction of caffeic acid (CAF) with cathepsin family receptor molecules. Docked orientation of (**A**) CAF (CID_637511), (**B**–**D**) 3D structure of cathepsin-K (PDB ID: 6QLM), hydrogen bonds between CAF with cathepsin-K, binding pocket of cathepsin-K. (**E**–**G**) 3D structure of Cathepsin-B (PDB ID:3AI8), hydrogen bonds between CAF with cathepsin-B, binding pocket of cathepsin B with CAF complex. Docking analysis was performed using Autodock tools (ADT) and Autodock v4.2 software.

Cathepsin is a cysteine residue based protease; ligand selection is based on its hot spot binding in substrate binding site of cathepsins family. These are the key factors for designing effective new ligand based inhibitors. Cathepsin-B structure showed arrangement of 18 amino acids long insertion from (Pro107-Asp124). In current study, orientations of the most potent inhibition of enzymatic activity of cathepsins-B and K were studied. It was found CAF docked similar binding regions with three-dimensional designed crystal structures of cathepsin-B and K (Figure 6A–G). The molecular modeling of CAF identified that a long hydrophobic pocket represents the potential binding site on the surface of cathepsin-K (−6.01) with two hydrogen bonds (Table 2), which is necessary for the peptide binding and excision (Figure 6B–D). The CAF docked the cathepsin-B in four hydrogen bonds, which at the site of hot spot of the receptor and made −4.43 binding energy (Figure 6E–G). Binding energy, ligand efficiency, and intermol energy were noted in Table 2.

## 3. Discussion

TCE showed remarkable osteogenic activity and improves bone strength. There is a possibility that the TCE contains active molecules responsible for the potential activity. Further refining and identification of constituents revealed the isolation of CAF. The structure was elucidated by inspection of ^1^H, ^13^C, DEPT, HMQC, and HMBC spectroscopic data (Table 1 and Supplementary Material) and compared with the literature values [21]. Whereas the ^1^H NMR spectrum of CAF (Table 1 and Supplementary Material) revealed the presence of aromatic resonances for an ABX system at σ_H_ 7.06 (d, J = 2.04 Hz), 6.80 (d, J = 8.16 Hz), and 6.95 (dd, J = 2.04, 8.16 Hz). In addition, a pair of doublet at σ_H_ 6.23 and 7.55 with coupling constants of 15.88 Hz was observed corresponding to two *trans*-olefin protons. By inspection of the ^13^C NMR spectrum (Table 1 and Supplementary Material), the obtained resonances were in agreement with the aromatic resonances at σ_C_ 127.82, 115.10,146.82, 149.47, 116.51, and 122.88, as well as two olefin carbons at σ_C_ 115.55 and 147.06 and a carboxyl resonance at σ_C_ 171.08, all of which were characteristic of caffeic acid. This study represents the first report on the isolation of CAF from *T. stellate*.

The obtained results establish the direct stimulatory effect of *T. stellata* metabolites on osteoblast differentiation. The potential stimulation of osteogenic properties of *T. stellata* metabolites has been proposed, but the molecular mechanism of this stimulation remains unclear. Natural products promote the osteoblast activity and suppress the osteoclast activity [22]. There are many studies from plants which showed the osteoblast activation and osteoclast inhibition processes [23,24,25]. The cytotoxic effects directly explained that the TCE was not toxic up to 100 μg/mL concentration. This biocompatibility on mesenchymal cells triggers us to investigate further on metabolites of *T. stellata*. The cytotoxic activity of CAF also explained its toxic properties and found nontoxic up to 10 μM concentration. The ALP and total protein were also reflecting this viability analysis. The results are comparable with study on medicinal plant’s (*Leonurus sibiricus*) ethanol extract and other herbal molecules, which showed osteoblast differentiation and suppress osteoclast differentiation as well as bone resorption in a mouse model [26,27,28,29]. One of the studies reported with osteogenic differentiation by the compound leonurine hydrochloride from *Leonurus sibiricus*. Some other studies similar to current study observed with BMP-2 transcription factor, which played key role in bone formation and osteoblast differentiation [23,30]. The presented results demonstrate that CAF from TCE stimulated mineralization in osteoblast by enhancing transcription factors and stimulating cathepsin-B and BMP-2 signaling. Likewise, CAF suppressed RANKL-induced osteoclast differentiation and resorption capacity of cells.

The guava fruit was also effective against osteoporosis according to Chinese medicine due to the presence of polyphenolic compounds and increased bone health [31,32,33,34,35]. The results showed similar properties as *Rumex crispus* extract in osteoblast differentiation through runt related transcription factor 2 and suppress the RANKL induced bone loss through suppressing the RANKL signaling [36,37]. Furthermore, it was observed that the transcription factors were enhanced to activate the osteogenesis [37]. It was found that CAF enhanced the mRNA levels of stimulatory transcription factors to induce osteogenesis. BMP-2 is the osteoblast-regulating factor that induced morphogenic protein involved in differentiation; mineralization and bone strengthen mediators, such as osteopontin and osteocalcin. The mineralization explained the loading of calcium in osteoblast cells improves the bone strength and vitamin-D signaling pathways [38]. The loading of calcium was noted for 21 days and found 50% elevated storage after the 14th day of differentiation. In both tested concentrations got a similar response in differentiation and ARS uptake. These results revealed that, the osteoblast differentiation and mineralization were dose dependent manner.

It is well established that, extracellular signal regulated kinase (ERK) and p38-MAPK are osteogenic mediated signaling pathways that regulate osteoblast markers in unique differentiation. The underlying mechanism of CAF was investigated on the osteogenic activity, the study was examined whether CAF from TCE regulates the BMP-mediated activation of signaling markers and down regulated the TRAP, MMP-9, and cathepsin-K markers. These bone factors considered as major transcription factors required for bone formation, associated with the regulation of osteopontin and osteocalcin mediators [37,38,39]. In addition, BMP-2-mediated Smad protein stimulation regulate the transcription of osteopontin and osteocalcin markers [40,41]. In this study, CAF synergistically upregulate the BMP-2 and showed reciprocal regulation of cathepsin-B with cathepsin-K, upregulate the osteocalcin as well as bone transcription factors (Figure 5 and Figure 6). These results suggest that the metabolic effect of CAF on BMP-2-dependent osteogenic to enhance the morphogenic protein mediated osteopontin and osteocalcin signaling axis for bone formation. It was showed that CAF treatments at low concentrations expressed an enhancement in differentiation and mineralization of osteoblast in both protein and mRNA level. Understanding the CAF effects on osteogenic markers, which delivers the outlined approach about the therapeutic potential for osteoporotic disorders. Furthermore, the regulations were confirmed by in silico docking analysis. The docking results revealed that, the structures of cathepsins-B and K are very similar, including their cleavage sites containing several “hot-spot” amino acids conserved among all types of cathepsins, along with Lysine 17 for cathepsin K and isoleucine 20 for cathepsin B receptors. The active site contains two histamine residues, which has high affinity towards substrate binding on receptors. These results revealed that CAF docked with cathepsin family receptors and plays a differential expressed ligand role in proteolytic protein osteogenic cell types.

## 4. Materials and Methods

### 4.1. General Experimental Procedures and Chemicals

^1^H-, ^13^C-, and 2D-NMR spectra were measured on an AVANCE 400 NMR spectrometer (^1^H-NMR: 400 MHz and ^13^C-NMR: 100 MHz, Bruker). Silica gel Column Chromatography (SCC) was performed on silica gel 60 (E. Merck, Darmstadt, Germany; 70-230 mesh). Reversed-Phase Silica Gel Column Chromatography (RPCC) was performed on a Cosmosil 75C18-OPN column (Nacalai Tesque, Kyoto, Japan; internal diameter = 50 mm, length = 25 cm, linear gradient: MeOH:H2O). Diaion HP-20 (Mitsubishi Chemical Co., Ltd. Tokyo, Japan). Pre-coated silica gel 60 F254 plates (E. Merck; 0.25 mm and 1000 μm in thickness) were used for Thin Layer Chromatography (TLC) visualization by spraying with p-anisaldehyde reagent and heated to 150 °C on a hotplate. High Performance Liquid Chromatography (HPLC) instrument used (Agilent, 1200 series, Germany) consisted of binary pumps, a PDA detector, and an auto sample injector, with a Chem satiation software module. The column was ZORBAX-SB-C18 (150 mm × 4.6 mm × 5 μm) (Agilent, Folsom, CA, USA). The mobile phase was in an isocratic mode using mixture of MeOH:H2O (45:55 *v*/*v*) with flow rate 1.0 mL/min. The volume of the samples injected into the HPLC system for analysis was set at 10 μL. Chemicals and reagents used were of analytical grade.

### 4.2. Plant Material

Aerial parts of *T. stellate* were collected from gardens of King Faisal University, Saudi Arabia (September 2016) and was identified by Dr. Mamdouh Shokry, Director of El-Zohria botanical garden, Giza, Egypt. Voucher specimen (9-16-Sept-TS) was deposited at the Herbarium museum of College of Clinical Pharmacy, King Faisal University, Saudi Arabia.

### 4.3. Extraction and Isolation

Shade-dried aerial parts of *T. stellata* (1.0 kg) were extracted three times with 70.0% methanol (10.0 L) by maceration at room temperature. The well-filtered extracts were combined and concentrated under reduced pressure to give an extract (103.0 g). The extract was partitioned with n-hexane (15.0 L) to give hexane fraction (74.0 g) and remaining mother liquor was concentrated to give (29.0 g) defatted extract. The defatted extract (25.0 g) was subjected to Diaion HP-20 column chromatography (1.0 kg) then it was eluted with water, 50.0% and 100.0% MeOH to obtain the fractions of water (5.0 g), 50.0% MeOH (12.0 g), and 100.0% MeOH (7.0 g). On the basis of the TLC patterns, the 50.0% MeOH-soluble fraction (12.0 g) was subjected to SCC (250.0 g, CHCl_3_:MeOH:H_2_O (15:6:1)(5.0 l)) then washing using 100.0% MeOH (2.0 l) to obtain seven sub-fractions (SubFr. 2-1 to 2-7). Sub-fraction 2-4 (1.3 g) was subjected to RPCC (100.0 g, using MeOH:H_2_O gradient elution) to yield six subtractions (SubFr. 2-4-1 (175.3 mg), SubFr. 2-4-2 (26.1 mg), SubFr. 2-4-3 (137.5 mg), SubFr. 2-4-4 (220.9 mg), SubFr. 2-4-5 (56.0 mg), and SubFr. 2-4-6 (330.7 mg)). SubFr. 2-4-4 (220.9 mg) was further fractionated on preparative TLC (CHCl_3_:MeOH:H_2_O (15:6:1)) the spot at R_f_ value of 0.59 was eluted (SubFr. 2-4-4-1) and was further purified by HPLC to give pure compound; caffeic acid (CAF) (Figure 1B,C).

### 4.4. Animals and Ethical Aspects

Male C57BL/6j mice 4-week-old were used and weighing between 15 and 20 gm body weight, acquired from the Animal Facility of College of Science of King Faisal University. Animals were housed in cages under standard conditions and room temperature (22 ± 2 °C) under 12 h alternating light/dark conditions. Animal acclimatization were followed by the standard procedure of animal experimentation, and protocols were approved by the Ethics Committee on animal use of the College of Science (No. 180123).

### 4.5. Culture of BM-MSCs

Primary bone marrow derived mesenchymal cells (BM-MSCs) harvested from bone marrow of male C57BL/6j mice using serum free culture medium [RPMI-1640; UFG, Yanbu, Saudi Arabia). Mice were euthanized by humane control and dissect the femora bones in aseptic conditions following [42]. This isolation procedure was used in all experiments such as osteoblast, osteoclast differentiation studies, respectively.

### 4.6. In Vitro Cytotoxicity Analyses

#### 4.6.1. MTT Assay

The cytotoxicity of different concentrations of TCE (10, 25, 50, 100, 250, and 500 μg/mL) and CAF (0.5. 1. 2, 5, and 10 μM) were tested in vitro in osteogenic medium. After the 7th day of TCE and the 7th and 14th day of CAF exposures, cell viability was evaluated by the MTT and the NR uptake assays [43] briefly, aspirate the supernatant after CAF treatments, the MTT reagent was added with fresh serum free medium and incubate for 3 h. The formation of formazan crystals were solubilized by adding 0.1 mL dimethyl sulfoxide, and the optical density (OD) was determined at 570 nm.

#### 4.6.2. Neutral Red Assay

The effect of CAF on neutral red uptake using osteogenic cells treated with CAF and cells were aspirate with 0.5 mL of neutral red (3-amino-7-dimethylamino-2-methylphenazine hydrochloride) solution (50 μg/mL). Then, it was incubated for 2 h at 37 °C. Thereafter, the supernatant aspirated with PBS, neutral red dye was extracted using 0.1 % glacial acetic acid, and the OD was determined at 540 nm [44].

### 4.7. Alkaline Phosphatase Activity

Alkaline phosphatase activity was determined at the 10^th^ day, using the Roy (1970) protocol [45], After CAF treatment, cell layers were scraped off using cell scrapper. Cell pellets were collected, and lysate was prepared by freeze–thaw method. After gently spinning down the cell debris, 20 μL supernatant from each sample were added to an assay mixture of p-nitrophenyl phosphate. Samples were quantified using 405 nm in Biotek elisa plate reader (Biotek Instruments Ltd., England). The specific activity of ALP was quantified by equilibrated total protein concentration.

### 4.8. Assays of Osteoblast Differentiation

Osteoblast differentiation was induced by addition of differentiation medium (DM), supplemented with 50 μg/mL ascorbic acid, 10 mM β-glycerophosphate, and 10 nM dexamethasone (Sigma-Aldrich, St. Louis, MA, USA). The negative group was kept without additives [42]. Osteoblast differentiation was assessed by mineralization of calcium using the protocols of ARS dye. Osteoblasts were treated with CAF at concentrations of 0, 5, and 10 μM for 21 days. CAF treated osteoblast cells were washed with ice-cold PBS buffer and fixed in ice-cold 10% formalin for 20 min. Then, 1% alcian blue solution was used for fixation. These sections were incubated for 8 min with ARS. Mineralized cell patches observed and counted using an image analyzing system EVOS (Life Technologies, Carlsbad, CA, USA).

### 4.9. Osteoclast Tartrate-Resistant Acid Phosphatase (TRAP) Activity Estimation

The effect of CAF was examined on osteoclast differentiation isolated according to previously described method [40]. After the RANKL induction, cells were treated with CAF at 5 μM and 10 μM for 7 days, and results were analyzed by TRAP activity assay [26].

#### Osteoclast Apoptosis 

The effect of CAF was evaluated on differentiation of pre-osteoclast cells isolated from bone marrow in mice. To generate osteoclasts, RANKL (150 ng/mL) was used for 4 days. Total TRAP activity was measured at an absorbance of 405 nm after treatment with Substrate (p-nitrophenyl phosphate) as described previously [26]. After treatment with CAF, results were obtained according to previously described protocol [46].

### 4.10. Gene Expression Analysis by Real-Time Reverse Transcriptase Polymerase Chain Reaction (qRT-PCR)

Total RNA extracted from CAF treated cells by the use of Trizol reagent (Invitrogen, Life Technologies, Grand Island, NY, USA). Extracted RNA were pretreated with DNAse I and quantified using Nanodrop (Thermo scientific, Waltham, MA, USA). Equiliberate the RNA concentration 300 ng/marker and used for complementary DNA preparation using SuperScript™III Reverse Transcriptase (Invitrogen, Waltham, MA, USA). Real time primer details were noted in Table 3. The relative mRNA quantification of cathepsin-B, cathepsin-K, MMP-p, BMP-2, TRAP, osteopontin, osteocalcin were evaluated by quantitative real time PCR (Applied Biosystems, Life Technologies, USA). Relative quantification of target genes were calculated using the 2−ΔΔCt method, as described by [47]. β-actin was used as an internal control.

### 4.11. Western Blot for Protein Marker Quantification

Protein quantification was done by Western blot analysis as described [48]. Briefly, CAF treated osteostromal cells such as osteoblast and osteoclast cells were washed with cold PBS. Cell pellets were collected by centrifugation and lysed by RIPA cell lysis buffer (Santa cruz biotechnology, USA). Cell lysates were obtained and total protein content in the supernatant was determined by the Bradford assay. It was eluted using SDS-PAGE, and the separated proteins were transferred to nitrocellulose membranes. Primary antibodies against cathepsin-B, cathepsin-K, MMP-p, BMP-2, TRAP, osteopontin, osteocalcin, and β-actin were diluted (1:1000; Cell Signaling, Danvers, MA, USA) Santa Cruz Biotechnology, Santa Cruz, CA, USA) at 4 °C overnight. Membranes were visualized using enhanced luminol-based chemiluminescent (ECL) substrate (Santa Cruz biotechnology, Santa Cruz, CA, USA).

### 4.12. Cytokine Estimation by ELISA

Production of tumor necrosis factor-α (TNF-α) and interlukin-10 (IL-10) in cell culture supernatant were evaluated. The BMP-2 and cathepsin-B were quantified in whole cell lysate of osteoblast treated with CAF using the manufacture protocol of Abcam validated kits (Abcam, Germany). Assays were performed on 96-well micro titer plates followed by manufacture instructions. The cytokine was quantified by the addition of chromogenic substrate solution (p-Nitrophenol). The reaction was measure at 450 nm. The standard curve was made in regression plot and sample concentrations were calculated in relation to the standard curve.

### 4.13. Computational Docking Analysis

The potential docking interaction was evaluated CAF against cathepsins-B and K were examined in silico using Autodock tools (ADT) v1.5.4 and Autodock v4.2 program (http://www.scripps.edu/mb/olson/doc/autodock). The respective chemical structure of ligand CAF (CID_637511), was retrieved from Pubchem database (http://www.ncbi.nlm.nih.gov/pccompound). The structures of cathepsin receptors were downloaded from the Protein Data Bank (PDB). The three dimensional structures of cathepsin-B (PDB ID: 6QLM) and cathepsin-K (PDB ID:3AI8) were retrieved from the Protein Data Bank; (http://www.pdb.org). The receptors were prepared by removing polar groups and non-amino acid residues using the protein preparation Wizard of PYMOL and python platform. The active sites of Cath-B and K were identified by Q-site Finder [49]. The competitive inhibition was studied on CAF complexed cathepsin protein family. Ligand docked to the receptor consider as rigid body, and receptor was considered as flexible factor. The ligand efficiency, intermol energy, and binding carbon numbers were recorded.

### 4.14. Statistical Analysis

The study was designed and evaluated in four independent experiments. The data values were expressed as means ± standard deviation (SD) (*n* = 4). Statistical analysis was conducted by student T test and one-way ANOVA regression plot compared with control group or treatment at different concentrations of TCE and CAF. All analyses were performed using Microsoft Excel office-10 and GraphPad Prism 7.0 (GraphPad Software Inc., San Diego, CA, USA), and statistical results are shown as the corrected p value (* *p* < 0.05).

## 5. Conclusions

Based on the current findings, it can be concluded that CAF treatment showed promising activation on the bone metabolism via osteoblast differentiation and bone maturation in C57BL/6j bone marrow derived cells. At low concentration of CAF treatments, it enhanced the mineralization and upregulated the osteogenic marker expression in mesenchymal cells. CAF induced the morphogenic protein of bone and cathepsin-B in bone derived precursor cells. On the other hand, CAF attenuated the osteoclast formation and differentiation through the inhibition of osteoclast markers such as TRAP, Cath-K, and MMP-9 in RANKL induced osteoclast cells. Moreover, it regulates apoptotic bone loss in osteoclast cells. These findings recommend, CAF derived for the first time from *T. stellata*, might be used as potential therapeutic alternative for the treatment of bone metabolic diseases.

## Figures and Tables

**Table 1:** NMR spectroscopic data of caffeic acid in CD_3_OD.

Position	δC	δH, mult. (J in Hz)
1	127.82	-
2	115.10	7.06, d, (2.04)
3	146.82	-
4	149.47	-
5	116.51	6.80, d, (8.16)
6	122.88	6.95, dd, (2.04, 8.16)
7	147.06	7.55, d, (15.88)
8	115.55	6.23, d, (15.88)
9	171.08	-

δC: chemical shift in ppm for ^13^C-NMR, δH: chemical shift in ppm for ^1^H-NMR; mult.: multiplicity; J in Hz: coupling constants in Hz; d: doublet, dd: doublet of doublet.

**Table 2:** Hydrophobic interaction of caffeic acid and amino acid residues of target proteins.

SL. No	Target Proteins	Binding Energy	Ligand Efficiency	Intermole Energy	Ligand Atoms (Ring)	Docked Amino Acid Residue (Bond Length)
1.	Cat-K	−6.01	−0.6	−6.55	C1-OH C1-OH	LYS`17/HZ2 (1.7 Å) LYS`181/HZ2 (2.3 Å)
2	Cat-B	−4.43	−0.44	−4.97	C1-OH C1-OH C1-OH C1-OH	ASN`222/OD1 (2.5 Å) THR`223/HN (2.2 Å) THR`223/HG1 (1.7 Å) ILE`20/O (3.2 Å)

Cat-K: Cathepsin-K, Cat-B: Cathespsin-B, LYS: Lysine, ASN: Asparagine, THR: Threonine, ILE: isoleucine.

**Table 3:** Real time PCR primer details.

Gene Name	Forward Primer	Reverse Primer
MMP-9	GTGCTGGGCTGCTGCTTTGCTG	GTCGCCCTCAAAGGTTTGGAAT
TRAP	GGTCAGCAGCTCCCTAGAAG	GGAGTGGGAGCCATATGATTT
OCN	AGCAAAGGTGCAGCCTTTGT	GCGCCTGGGTCTCTTCACT
OPN	ACATCCAGTACCCTGATGCTACAG	TGGCCTTGTATGCACCATTC
Cath-K	GCCAGGATGAAAGTTGTATG	CAGGCGTTGTTCTTATTCC
Cath-B	GGTTGCAGACCGTACTCCAT	GGAACTGCATCCAAAATGCT
BMP-2	TGCACCAAGATGAACAGC	GTGCCACGATCCAGTCATTC
GAPDH	GTATTGGGCGCCTGGTCACC	CGCTCCTGGAGATGGTGATGG

## Supplementary Materials

The following are available online at https://www.mdpi.com/2223-7747/9/11/1610/s1, Figure S1–S8: 1 D- and 2 D-NMR spectroscopic data of CAF.
