# Supplementary material for: Biomolecule from Trigonella stellata from Saudi Flora to Suppress Osteoporosis via Osteostromal Regulations
Source: Plants (Basel). 2020 Nov 20;9(11):1610. doi: 10.3390/plants9111610 (PMC7699612; doi:10.3390/plants9111610)
Supplement: Supplementary file 1 [file plants-09-01610-s001.pdf]

Supplementary Materials

Figure S1–S8: 1 D- and 2 D-NMR spectroscopic data of CAF

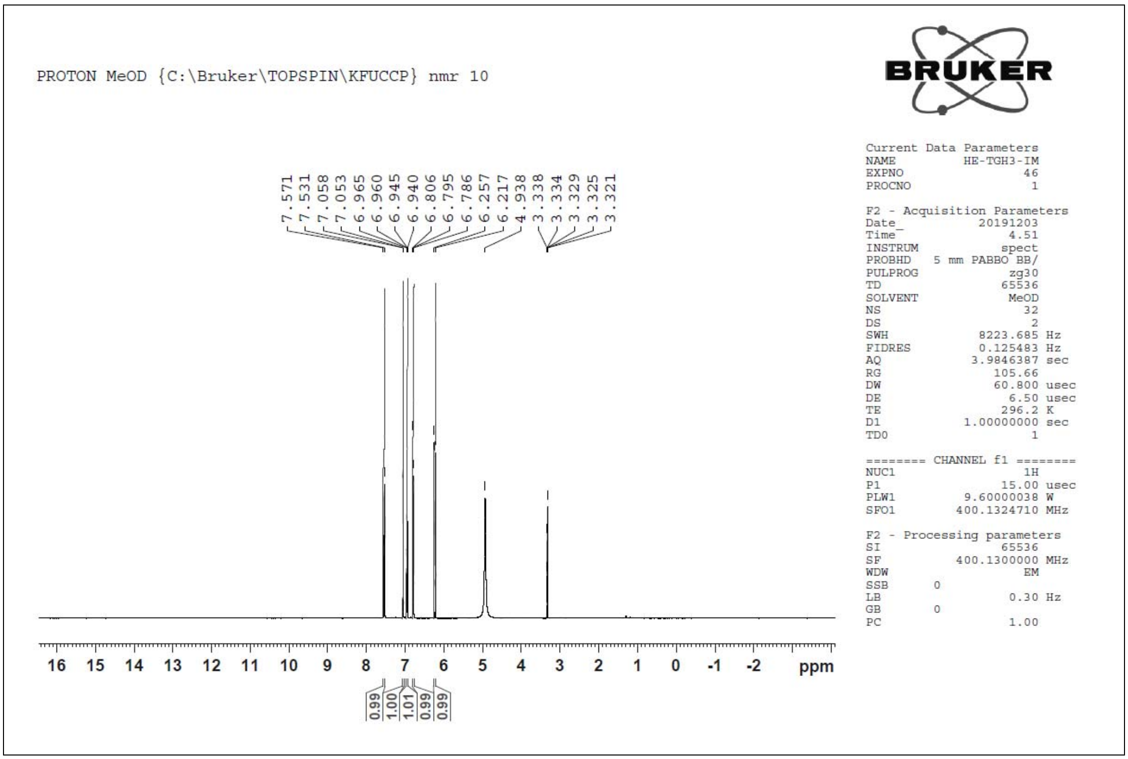

Figure S1. ^{1}H-NMR spectrum of CAF (CD3OD).

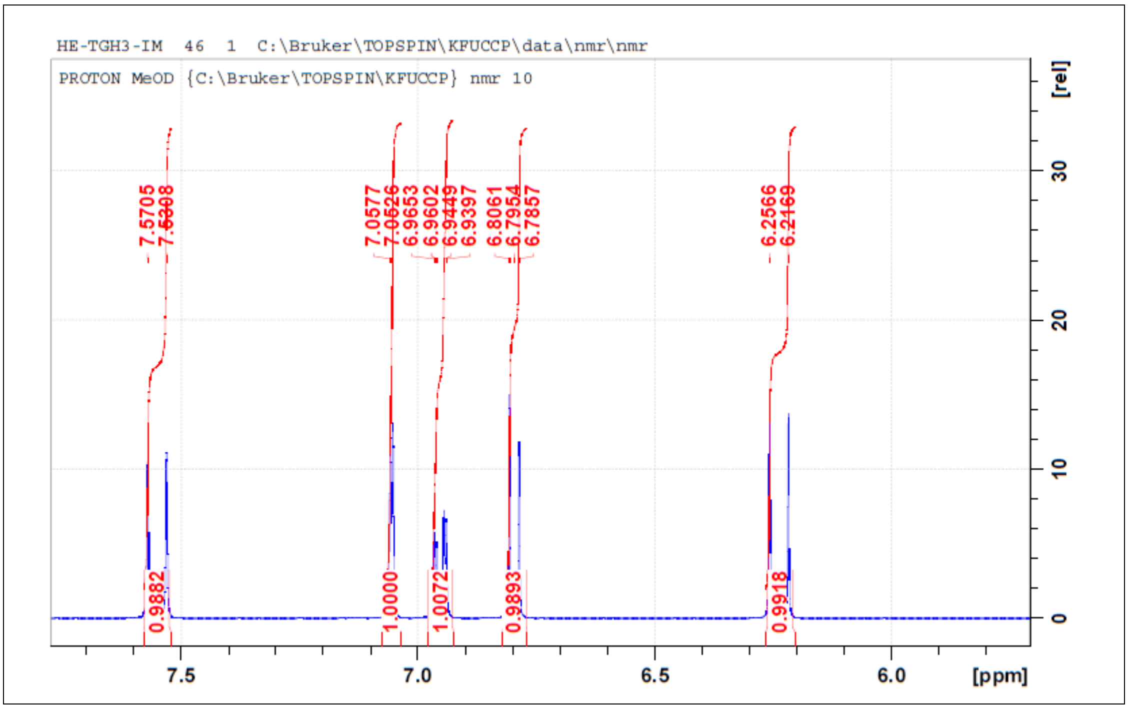

Figure S2: Expanded ^{1}H-NMR spectrum of CAF (CD3OD).

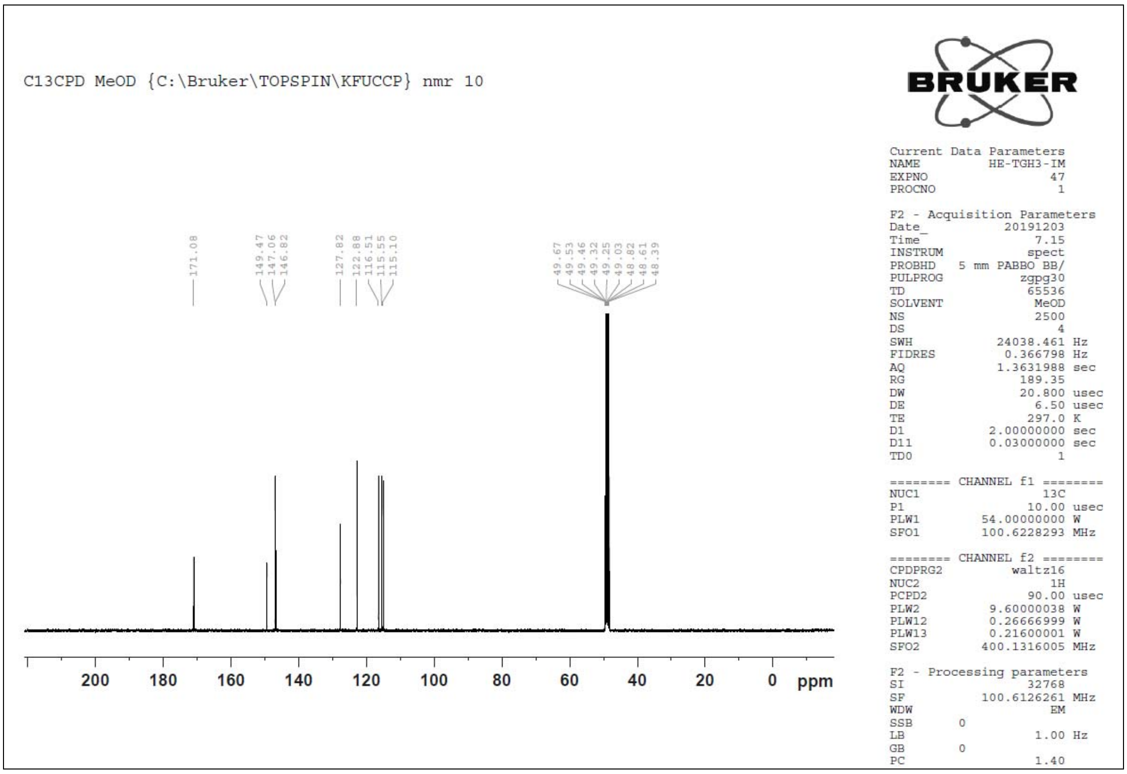

Figure S3: ^{13}C-NMR spectrum of CAF (CD3OD).

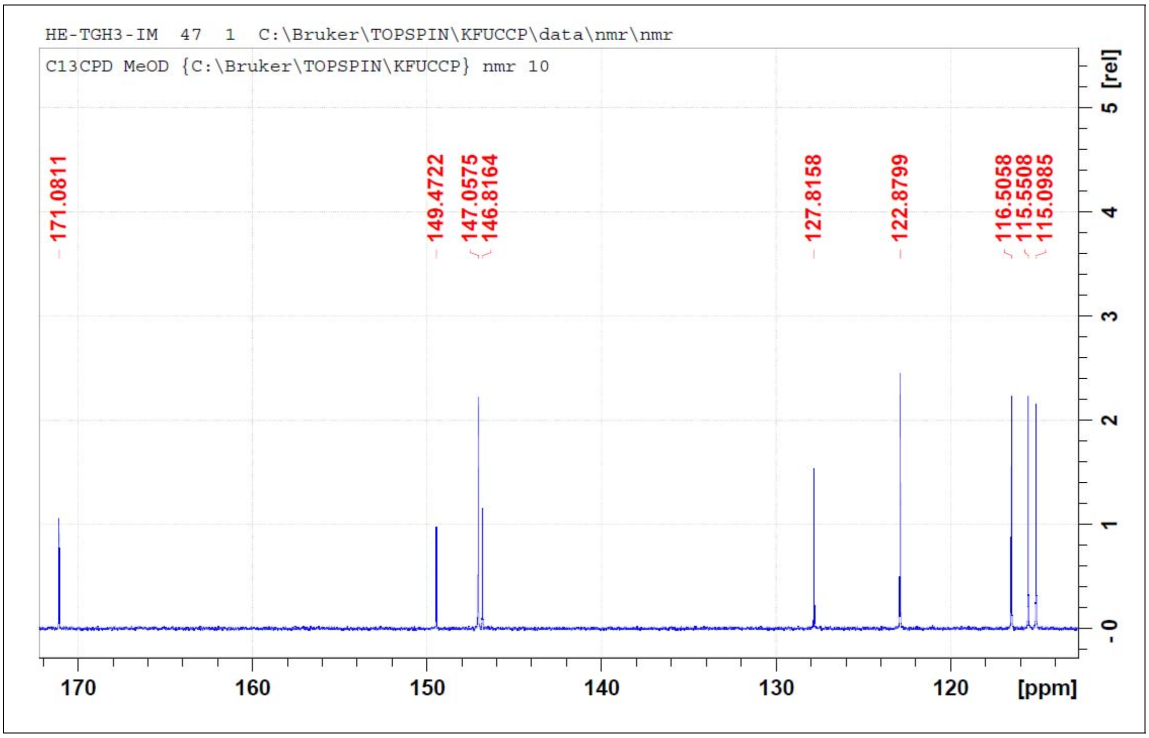

Figure S4: Expanded ^{13}C-NMR spectrum of CAF (CD3OD).

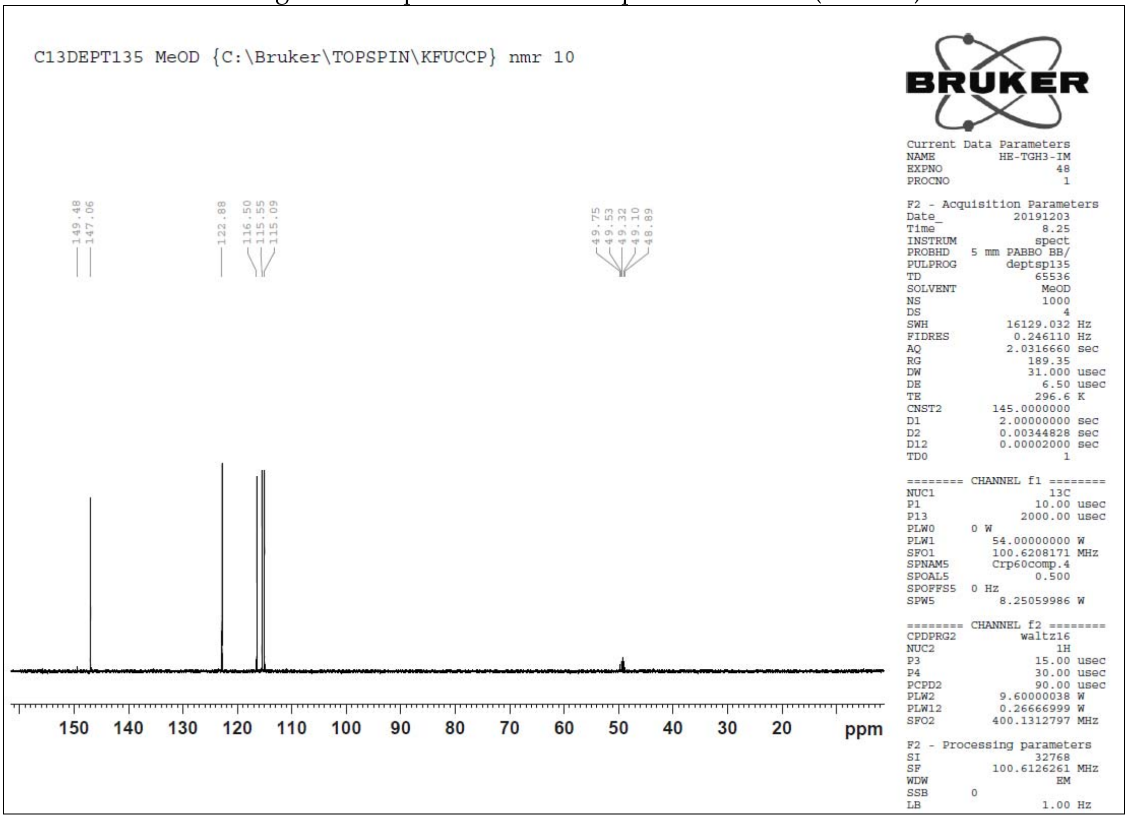

Figure S5: DEPT spectrum of CAF (CD3OD).

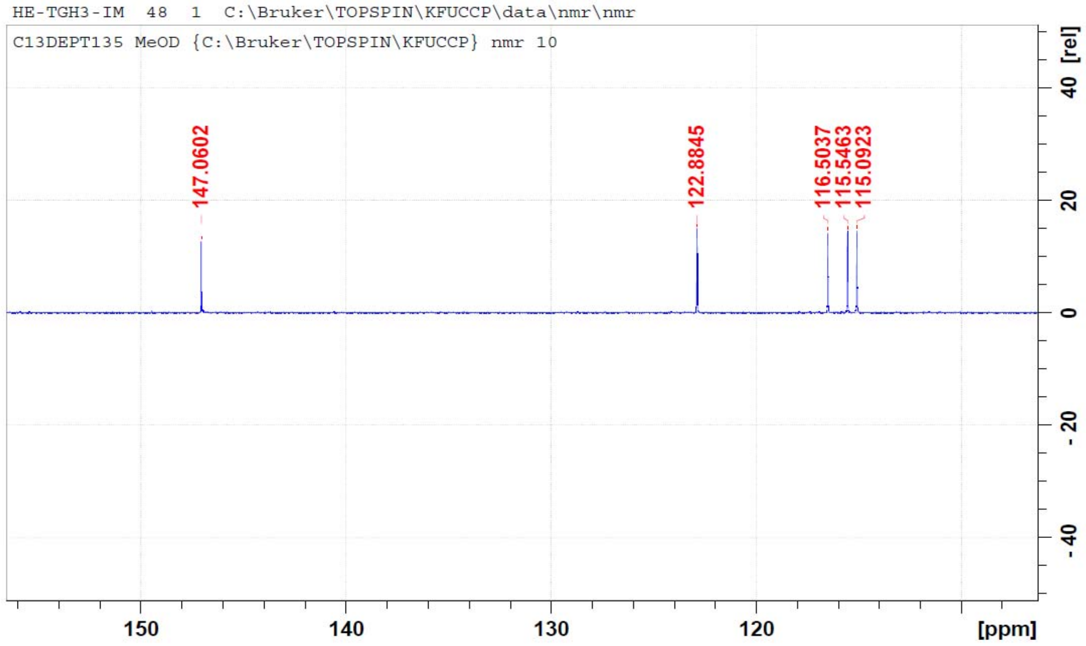

Figure S6: Expanded DEPT spectrum of CAF (CD3OD).

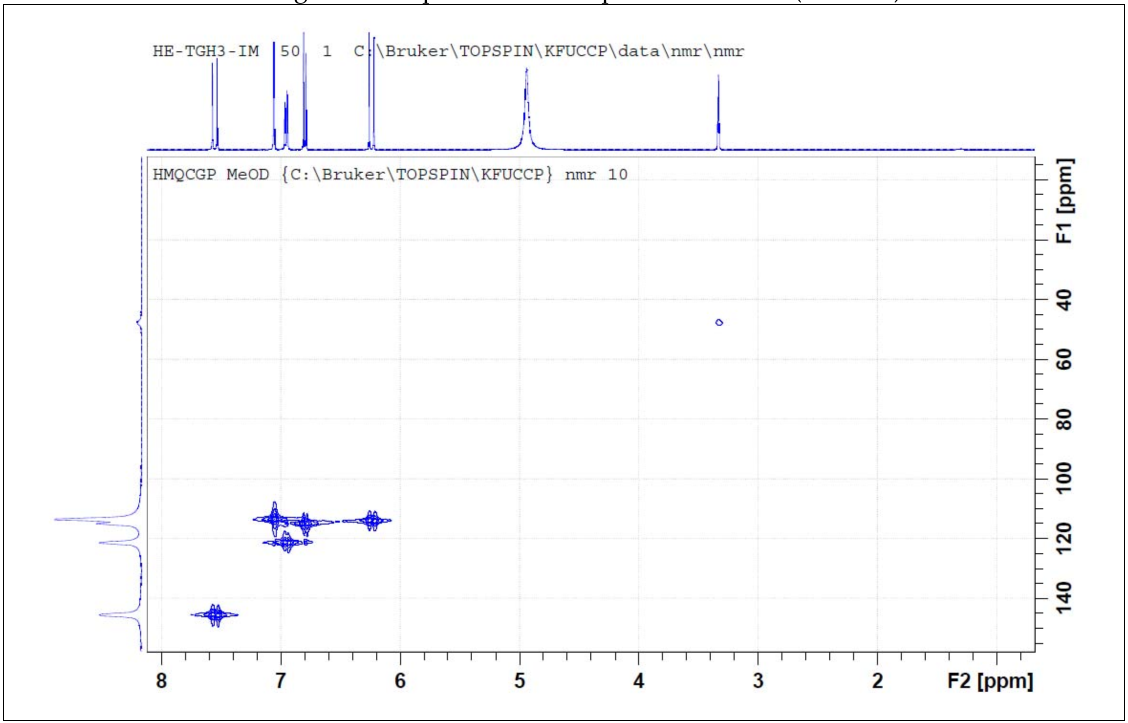

Figure S7: HMQC spectrum of CAF (CD3OD).

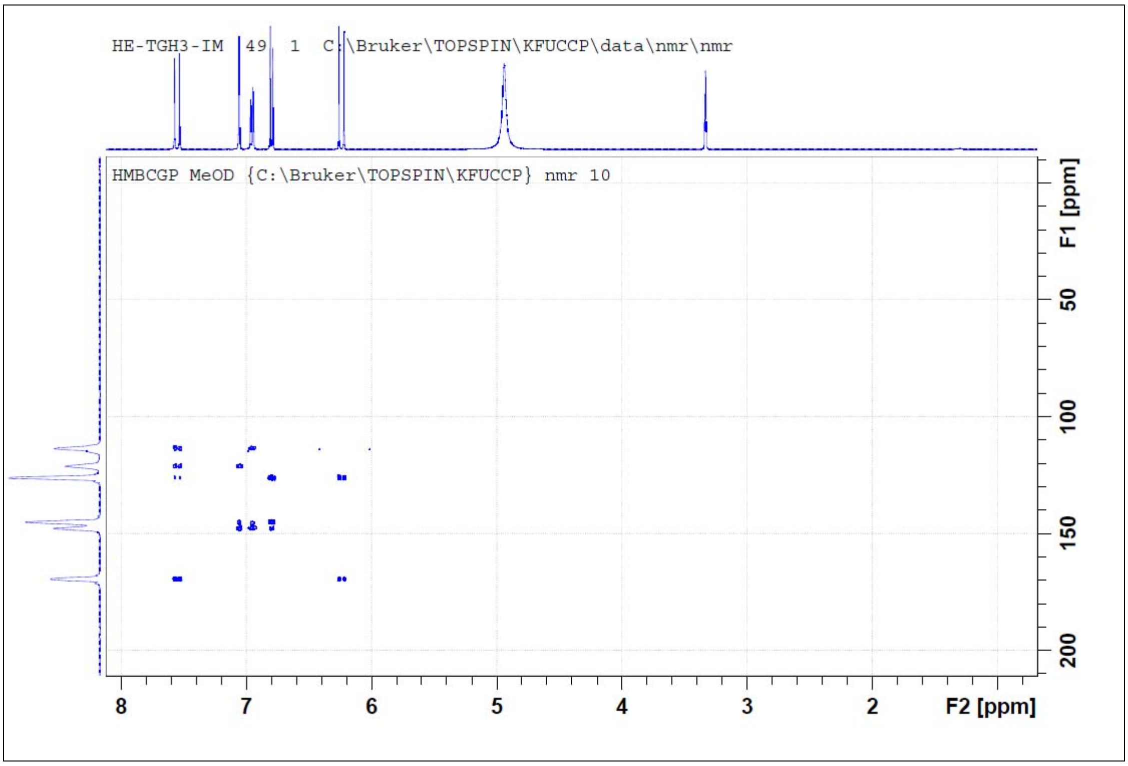

Figure S8: HMBC spectrum of CAF (CD3OD).
